# Knowledge-Driven Event Extraction in Russian: Corpus-Based Linguistic Resources

**DOI:** 10.1155/2016/4183760

**Published:** 2016-01-05

**Authors:** Valery Solovyev, Vladimir Ivanov

**Affiliations:** Kazan Federal University, Kremlevskaya Street 18, Kazan 420008, Russia

## Abstract

Automatic event extraction form text is an important step in knowledge acquisition and knowledge base population. Manual work in development of extraction system is indispensable either in corpus annotation or in vocabularies and pattern creation for a knowledge-based system. Recent works have been focused on adaptation of existing system (for extraction from English texts) to new domains. Event extraction in other languages was not studied due to the lack of resources and algorithms necessary for natural language processing. In this paper we define a set of linguistic resources that are necessary in development of a knowledge-based event extraction system in Russian: a vocabulary of subordination models, a vocabulary of event triggers, and a vocabulary of Frame Elements that are basic building blocks for semantic patterns. We propose a set of methods for creation of such vocabularies in Russian and other languages using Google Books NGram Corpus. The methods are evaluated in development of event extraction system for Russian.

## 1. Introduction

Automatic event extraction is an important task in knowledge acquisition step. This task needs a lot of manual work both in corpus annotation and in vocabulary population and extraction pattern construction. Current state of the information extraction (IE) domain can be described as follows. Modern enterprise systems are developed using a knowledge-based (KB) approach that requires an expert for construction of patterns for extraction structured objects from text. These patterns have high precision and good recall values but demand a vast amount of manual work of experts. Few companies follow this way: ONTOS (http://www.ontos.com) [1], RCO (http://www.rco.ru/) [2].

An alternative, a data-driven approach, relies on machine learning methods and demands manual work for annotation of corpora. Amount of manual work is comparable to the KB approach, because annotated corpora should contain thousands of labeled objects and relations. That is why development of methods that require less effort is an important problem in information extraction.

Both knowledge-driven and data-driven approaches have drawbacks. Former one needs a lot of manual work of high quality experts and is complicated when an existing set of extraction rules should be ported to novel domain. Latter one requires annotation of large corpora, comparable amount of manual work, and its results are harder to interpret. That is why many attempts have been done to combine two approaches in a single system and to avoid drawbacks of both [3–7]. A survey on hybrid systems could be found in [8]. Due to complexity of architecture, a combination of knowledge-driven and data-driven approaches may appear in many different ways. The hybrid approach proved to be very efficient in extraction of events when event triggers are represented as nouns (e.g., agreement and construction) derived from verbs (e.g., to agree and to construct) [6]. A well-known approach that uses CRF in most cases fails to identify such events. In [6] authors have proposed several ways for including expert knowledge into CRF model. First, they use Wordnet to get comprehensive part-of-speech information. For instance, a word “war” in Wordnet has both verb and noun attributes. Second, they use Semantic Role Labeling [4] to get semantic roles of arguments of an event trigger represented by either verb or noun. Third, it is important to define a separate set of extraction rules aimed at extraction of events represented as verbal nouns. These extraction rules extensively use morphology (e.g., verbal noun usually has “-tion,” “-ion,” “-ing,” and “-ed” suffixes), syntactic constructions (such as pronouns “during,” “after,” and “before” followed by an event trigger). The TempEval-2010 Evaluation (http://timeml.org/tempeval2/) has shown a significant growth of *F*_1_-measure from 76.87 (for CRF only) to 94.47 (for CRF + WordNet + Semantic Role Label + Rules). It is apparent that expert knowledge may improve results in addition to the use of machine learning methods.

In a number of studies it has been noted that a purely data-driven approach does not provide good results with respect to the extraction of the organizations and persons (event participants); for example, in [5] extracting information from texts of the German Parliament has been studied. Better results can be achieved by manual filling of a list of organizations. Similar problems and approaches are found in other domains. For instance, in [3] to extract biomedical information about proteins and their interactions authors combine data-driven approach using vocabularies of protein names. Initial lexicons are expanding with simple generalizations of manually created rules. To extract protein interactions in their study, authors use a similar hybrid method consisting in learning extraction rules (and their generalization in a straightforward way). In another study on biomedical problems [7] a hybrid approach has been developed in a slightly different manner. Event triggers have been defined by machine learning. Events are described by rules, wherein the rules themselves are generated by parsing algorithms.

Generally, the use of parsing leads to hybrid systems. On the one hand parsing uses linguistic patterns or hierarchies of dependencies components, and, on the other hand, it is usually trained on corpora, which allows collecting statistics and treating the approach as a data-driven one. In general, typical hybrid systems combine statistical methods with linguistic and ontological resources. This fact confirms the importance of such resources, regardless of the underlying approach to the creation of event extraction systems.

Hybrid systems typically require less manual effort than knowledge-driven approach, due to the fact that some domain knowledge is obtained by statistical methods. And they also require less amount of annotated data than data-driven approach, due to the fact that some of the important information is encoded in the rules. However, it should be noted that the hybrid approach is not a one-fits-all solution. Combination of the advantages of knowledge-driven and data-driven approaches sometimes leads to combination of the disadvantages as well. The success of a hybrid system depends on a finely balanced combination of different techniques and methods. The hybrid approach is largely the art.

A problem specific for IE in Russian is a lack of resources (vocabularies, corpora, and methods for natural language processing) that form an environment for creation of IE systems. More than several hundreds of articles study IE in English and other European languages. Tens of systems have been created. In Russian since the domain does not attract many researchers, the IE methods are not studied well. Most of works are restricted to named entity recognition [9]. Commercial systems are not described and have never been tested at the international level. One of the main efforts in IE is the Message Understanding Conference (MUC (http://www.itl.nist.gov/iaui/894.02/related_projects/muc/proceedings/muc_7_toc.html)) that has been held since 1987 till 1997 with support of DARPA (Defense Advanced Research Projects Agency). The conference stated a set of standard approaches to measuring quality of an IE system.

The next step was the Automatic Content Extraction initiative (ACE (http://www.itl.nist.gov/iad/mig/tests/ace/)), proposed in 1999. The initiative resulted in creation of corpora for IE systems evaluation. In contrast to MUC, ACE focuses on entity extraction (i.e., words that mention entity) and not just words that name some entity. ACE proposes to extract entities, relationships, and events which are more complex tasks. Event extraction task started with 8 event types (33 subtypes) from a wide sources such as newswire, broadcast conversation, and weblogs. Before moving further we need to define important terminology, proposed in ACE. The ACE program defined the following terminology for event extraction task:

*Trigger*. The word most clearly expresses an event's occurrence.

*Argument*. An entity mention, a time expression, or value plays a certain role in the event instance.

*Event Mention*. A phrase or sentence has a distinguished trigger and arguments. 

In the paper we describe resources necessary in KB-systems: linguistics databases and vocabularies such as thesauri and frames. An event is defined as a set of frame structures that include obligatory and optional arguments, their roles, and types. An event extraction system maps each sentence with some frame from the set and fills the arguments and roles with values and entities found in text. A frame language is aimed at using subordination models of verbs and takes into account specific features of Russian language. A mapping algorithm extracts verb and noun triggers (both single word and multiword), finds entities with their heads, and maps each head to a certain valency of a trigger using linguistic and ontological knowledge. A typical scenario for event extraction includes following steps:

- finding sentences that contain an event trigger;
- finding mentions of event participants that are expressed by actants of the trigger word.

Alternative scenario is a linear template matching algorithm. In this scenario a template matches parts of a sentence (i.e., strings of words) and does not use frame structure. We argue that nonlinear templates that use frame structures are appropriate in event extraction from Russian text.

*Frame-Based Nonlinear Templates and Event Coding*. Nonlinear template is a nonempty set of basic rules, each having the following representation: trigger-participant. Each rule is intended for extraction of an event participant (we treat a participant as a word that represents an event argument and fills a valency of a trigger). A sample template with two triggers is provided below:

 покупать (buy)
 
{
 покупать - Artifact, Винительный  падеж (Accusative), -  ;
 покупать - Seller, Родительный  падеж (Genitive), у (from) ;
 покупать - Buyer, Именительный  падеж  (Nominative), -  ;
 покупать - Price, Дательный  падеж (Dative), по (by) ;
 покупать - Place, Предложный  падеж (Locative), в (in) ;
 
}
 продавать (sell)
 
{
 продавать - Artifact, Винительный  падеж (Accusative), -  ;
 продавать - Buyer, Дательный  падеж (Dative), -  ;
 продавать - Seller, Именительный  падеж (Nominative), -  ;
 продавать - Price, Дательный  падеж (Dative), по (by) ;
 продавать - Place, Предложный  падеж (Locative), в  ;
 
}

This sample shows that in the same event triggers may have completely different subordination models. For example, buyer is expressed by nominative case as well as by dative case without preposition; seller is expressed by genitive case with preposition as well as nominative case. This sample demonstrates that a developer of an event extraction system has to solve the following tasks:

- to find triggers for each type of event;
- to find a set of models that express a certain pair (trigger-participant) in a sentence;
- to discover frames that describe a given event type and its participants.

Due to a large number of verbs and their combinations with cases and prepositions, manual development of a KB-system with nonlinear templates appears to be an impossible task. Additional issue is that an expert is not aware of distribution (in a given fraction of texts) of a certain template (except the rule of thumb that each template he/she defines will cover some small fraction of text). However, distribution of a template part allows focusing on more frequent cases and ignoring less frequent ones.

To support our argument we compared two simple implementations of event extraction systems for the Hewlett-Packard Company [10]. The comparison showed that frame-based templates work better in terms of precision and recall than linear templates. Even though nonlinear templates need sophisticated processing of text, it is much faster to define them, if a developer has additional resources (such as a vocabulary of subordination models, thesauri, and parser). Thus, we decided to follow the lines of the nonlinear templates for KB-system.

We pay less attention to event coding in this paper, because the main motivation behind this work is to facilitate an existing event extraction system for Russian that has been developed and evaluated in [10]. The system is aimed at extraction event types described in ACE [11].

According to [11], the central concept of event extraction is an indicator word (usually a separate verb) denoting a certain type of event. We consider the case when an indicator is the main verb (or predicate) that controls all participants of event in a sentence (i.e., participants of the event act as syntactical arguments of the predicate). An extraction template in this case will reflect a model of control for the predicate (verb).

**Figure 1:**
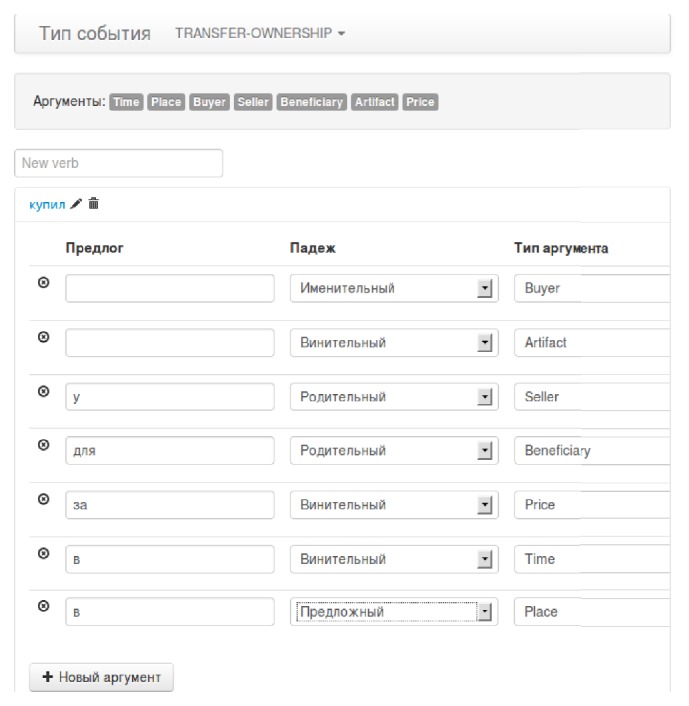
A simple user interface for definition of event extraction templates.

We developed a user interface that allows defining nonlinear extraction templates (or rules). A sample rule is shown in Figure 1. Here we can add verbs and fill their subordination model for a given type of event. New verbs can be added and models for existing verbs can be edited by clicking on it. After that, simple tabular interface appears. It shows one argument of model of control for corresponding verb per line. Expert can easily set preposition (if any), grammatical case of the argument and select argument type (from predefined list); but it becomes harder to define all the prepositions and cases as the number of event types and verbs grows up.

Thus, here we focus on automatic building of set of linguistic resource, that will facilitate constructing and exploration of nonlinear templates in an existing knowledge-based event extraction system.

## 2. Materials and Methods

Any approach to knowledge-based event extraction needs construction of linguistic resources necessary for sophisticated preprocessing of input text, trigger discovery, and connecting trigger with participants of the event. We describe these linguistic resources in the following subsections.

### 2.1. Existing Linguistic Resources for Event Extraction

Semantic analysis of input text is an essential step in knowledge-based event extraction. Semantic processing procedures such as tagger, parser, and word sense disambiguation need a set of linguistic resources. Therefore, our first goal is construction of appropriate resources in Russian.

Manual construction of the vocabularies is a very time-consuming task, but in most cases this is the only way to describe a certain part of language. Fortunately, several necessary vocabularies and corpora have been already developed. We will describe all those existing vocabularies first as they are of high importance in constructing other semantic vocabularies and resources. The list of existing resources we use in event extraction includes

- Russian Open Corpus (ROC or OpenCorpora) and corresponding morphology vocabulary;
- a part of Russian National Corpus (RNC) with disambiguated morphological labels;
- Russian treebank: a SynTagRus corpus with dependency trees of few thousand sentences;
- a thesaurus of Russian language: RuThes-lite.

Further we will describe each resource and its purpose in event extraction.

#### 2.1.1. PoS-Tagging: OpenCorpora and Russian National Corpus

The two main corpora in Russian are RNC [12] and OpenCorpora [13]. They have slightly different tag sets, and both use Zaliznjak's model of grammatical categories [14]. Each tag describes not only a part-of-speech of a given word, but other grammatical information pieces such as a case, gender, and number. The ROC-based morphological vocabulary contains about 390,000 lexemes and over five million word forms. We use this vocabulary if no disambiguation is required.

An available part of the RNC contains one million words with correct PoS-tags. This data is proposed for comparative evaluation of PoS-taggers for Russian. Authors evaluated performance of seven taggers including HunPos, Stanford, PoS-tagger, OpenNLP implementation of MaxEnt Markov Model, Tiered Conditional Random Fields (TCRF), and two baselines. The TCRF classifier trained on the RNC data shows the best performance of a PoS-tagging in terms of an accuracy measure (93–95%). Result of the PoS-tagging phase is crucial in event extraction, because a PoS-tag label of a word is used in the next step: a dependency parsing phase.

#### 2.1.2. Dependency Parsing: SynTagRus and MST-Parser

Building a dependency parser is important step, because it allows extracting syntactic dependencies between words. It is especially important in Russian due to a free word order in a sentence. Despite a plethora of researches in dependency parsing in the past decade, the dependency parsing in a particular language needs a syntactically annotated corpus.

In 2012, the RuEval-12 initiative (http://otipl.philol.msu.ru/~soiza/testsynt/) has evaluated parsers for Russian. Even though there was no standard representation for the dependency tree shared by all parsers, the gold standard (consisting of about 800 sentences) for evaluation was developed. Best parsers show quite high values of *F*_1_-measure (94–96%).

Most parsers for Russian are either closed-source projects or poorly documented and, therefore, cannot be included in an event extraction pipeline. Though, this situation has a notable exception: MaltParser for Russian, trained on 630,000-word corpus and presented in [15]. However, their model is not really fast and uses a quite different tag set, that is, MULTExt-East [16].

Thus, we decided to develop and independently evaluate a dependency parser for Russian. The parser is implementation of well-known MST-Parser algorithm [17]. The parser was trained on a set of 8,000 sentences from the SynTagRus corpus which is a part of RNC [12]. This set is published on the RNC website (http://www.ruscorpora.ru/search-syntax.html).

In the evaluation of the parser we have run a standard tenfold validation scheme. PoS-tagging was derived from the TCRF classifier described above. A performance of the parser was as good as 85% according to an unlabeled attachment score measure.

A parsing phase discovers syntactic dependencies from a raw sentence. Each dependency is a pair of words: “head” and “dependent.” If the sentence contains a trigger of an event, then dependencies where trigger plays the “head” role may indicate some participants of the event. In the simplest case, trigger (say, a verb) has a direct object considered as a participant. In a more complex case, a trigger superordinates a preposition phrase that mentions an event's argument or participant. These “possible” dependencies are well-known as valences and had been described by Tesnière and Fourquet [18]. A set of valences for a given word is a subordination model.

### 2.2. Vocabulary of Subordination Models

The vocabulary of subordination models is really hard to produce manually, but it is important in event extraction. Such vocabulary allows a developer to focus only on trigger's valences. The vocabulary would significantly reduce a set of all possible combinations of pairs “trigger,” “a case of a dependent” (e.g., “to buy,” “accusative”) and triples “trigger” - “preposition” - “case of a dependent” (e.g., “to buy” - “in” - “Locative” ).

Recent research has been carried out in the area of generating subordination models for Russian verbs. Kochetkova and Klyshinsky [19] use a web corpus. They proposed a method for automatic generation of vocabulary of subordination models for verbs and prepositions. The method works on a lexical level, that is, using the information about case of nouns controlled by verb through specified preposition. The extraction of verb(-preposition)-noun dependencies is done with a set of finite automatons. Dependency parsing was not used in corpus processing.

Resulting dataset was filtered to exclude grammatical ambiguity, rare word combinations that are not allowed in Russian grammar. Unfortunately, the vocabulary is not available. Other works have an insufficient vocabulary size that prevents using such vocabularies in a computer system. Existing treebanks of Russian language also have insufficient corpus size for automatic generation of a more or less complete verbal subordination vocabulary.

Main problem in automatic extraction of subordination vocabulary is ambiguity. In the case of subordination, ambiguity in Russian language is different from ambiguity in English. To reduce noise in the resulting vocabulary ambiguous part of text should not be processed at all. A corpus for such extraction should have a huge size. We are not aware of a corpus of an appropriate size with dependency annotations, except the Google Books Ngram Corpus (GBNC).

#### 2.2.1. Google Books Ngram Corpus

This corpus describes how often words and phrases were used over a period of five centuries, in eight languages; it reflects about 6% of all books ever published. Russian subset of GBNC contains 67,137,666,353 tokens extracted from 591,310 volumes [20] mostly from past three centuries. Each book was scanned with custom equipment and the text was digitized by means of OCR. Only those *n*-grams that appear over 40 times, are included into the dataset.

The original GBNC dataset contains statistics on occurrences of *n*-grams (*n* = 1,…, 5) as well as frequencies of binary dependencies between words (http://books.google.com/ngrams/). These binary dependencies represent syntactic links between words from Google Books texts. The GBNC stores all statistics on a year-by-year basis; each data file contains tab-separated data in the following format (http://storage.googleapis.com/books/ngrams/books/datasetsv2.html):

 
n-gram, year, match_count, volume_count.

Latest version of GBNC introduced syntactic annotations: words were tagged with their part-of-speech, and head-modifier relationships were recorded. An accuracy of unlabeled attachment for Russian dependency parser reported in [20] is 86.2%. Further, we extensively use these relationships for generating of new vocabularies.

#### 2.2.2. Google Books' Dataset Preprocessing

The main preprocessing step that allows using the GBNC is enrichment of the corpus with morphological information. We have preprocessed the original dataset in a special way. First, for each dependency 2-gram (the same step for each 3-gram), we have collected all of its occurrences on the whole dataset and added up all “match count” values since 1900. Aggregated dataset consists of pairs (*n*-gram, count), *n* = 2,3. This step also lowered case of letters in each *n*-grams in order to decrease variability.

In the next step we assigned each word in a 1-gram dataset with a PoS-tag and morphological features. For this purpose we used an OpenCorpora morphological vocabulary. This resulted in a dataset that has the following format:

 
n1, match_count, pos, lemma, gram.

For ambiguous words we will obtain several records.

 
n1, match_count, pos, lemma, gramA;
 
n1, match_count, pos, lemma, gramB;
 
…

In all such cases, we omitted rows with alternative grammatical interpretations from the dataset, because taking these records into account adds a lot of noise. We denote a morphologically enriched dataset as m-GBNC.

#### 2.2.3. Subordination Vocabulary for Russian Verbs

When constructing the vocabulary of subordination models, we focus on subordination models for Russian verbs. Let us briefly describe a technique we use to generate a vocabulary of subordination models. First, we capture all pairs (head, dependent) with PoS-tag of the “head” part equal to “VERB” and with a certain grammatical case of the “dependent” part, say, “gent” for the genitive. Finally, we group all these pairs by “lemma” (different forms of the verb share the same “lemma”) and count the number of records in each group and add up match_count values. Basically, we run the following SQL-query against the m-GBNC dataset (“dep_bigrams” is a name of the table with enriched dependencies data) (see Algorithm 1).

In Algorithm 1 we have six aggregation (sum) functions (one for each grammatical case, e.g., “loct” for the locative, “nomn” for the nominative). Each aggregation function in the query calculates total amount of dependency links between verbs given a lemma_id and arbitrary word forms in a certain grammatical case. We apply the same technique when generating a model for subordination of a preposition in the 3-gram dataset. These two types of queries differ only in the WHEN-conditions and the GROUP-BY operator that includes additional restriction on the second word (that has to be a preposition) in a 3-gram.

The result of this step is a set of merged records. Each record descries a basic part of subordination model for some verb and has the following format:

 
VERB,
   
Nominative_COUNT,
   
Genitive_COUNT,
   
Dative_COUNT,
   
Accusative_COUNT,
   
Instrumental_COUNT.

We eliminated the locative case, because in Russian it requires a preposition. This dataset contains about 24 thousand rows (one row per verb). If the verb subordinates a preposition then record has different format:

 
VERB, PREP,
   
Nominative_COUNT,
   
Genitive_COUNT,
   
Dative_COUNT,
   
Accusative_COUNT,
   
Instrumental_COUNT,
   
Locative_COUNT.

This dataset contains about 51.5 thousand rows (a verb + preposition per row). Having such a vocabulary a developer of event extraction system can explore models for event triggers, represented as verbs. However, the list of event triggers itself might be incomplete. In the next section we discuss an algorithm for event trigger extraction.

### 2.3. Vocabulary of Event Triggers

If a developer of an event extraction system is interested in a certain event type, then it is necessary to produce a vocabulary of event triggers for the type (or trigger list). This trigger list may include

- synonyms (e.g., buy and purchase),
- associated words (e.g., buy and sell),
- related words with different part-of-speech tags.

We propose an approach for generation of a list of event triggers. The approach uses GBNC dataset in the following fashion. First, we extract all 3-grams (*X* *c* *Y*), the *X* and *Y* have the same part-of-speech (either noun, verb, or infinitive); *c*, the middle word in a 3-gram, is a conjunction (either or or and).

Statistics of the whole derived dataset are provided in Table 1.

Similar words are not necessarily appearing in the same 3-gram. However, we suppose that similar words will induce similar sets of 3-grams. A simple bootstrap algorithm that uses this idea is presented below.

(1) Let *S* be a set of seed trigger words *S* = {*s*_*i*_}.
(2) For each *s*_*i*_ extract 3-grams (*s*_*i*_ *c* *X*) and (*Y* *c* *s*_*i*_), where *c* ∈ *C*, *C* = {AND, OR}.
(3) Get the most frequent words that appeared in positions of *X* and *Y* and show them to an expert.
(4) Appropriate trigger words *s*_*k*_′ are selected by the expert and then included in the seed set *S*. If expert does not select new seed triggers, the algorithm is finished; else go to step (1).

A separate task in event extraction is attaching an event participant to the trigger within a certain role (or a slot). This is especially important when an event trigger is represented as a composite of few syntactically related words. In such case, participants of an event may be attached to different parts of a complex trigger [21]. Here we will discuss a slightly simple case when an event trigger is a single verb or a noun.

### 2.4. Vocabulary of Frame Elements

Given a verb that represents an event trigger, each pair (verb, case) or each triple (verb, preposition, and case) should be associated with a semantic role (e.g., “seller,” “buyer,” “goods,” and “place”). In this step, a developer of event extraction system needs a FrameNet-like resource that describes all possible roles for the verb. Automatic generation of FrameNet is nearly an impossible task. However, we propose a method for automatic extraction of frame parts that are of high importance in FrameNet construction.

#### 2.4.1. FrameNets and Russian FrameNet

FrameNet and similar resources (VerbNet and PropBank [22]) play important theoretical and practical role. FrameNet facilitates semantic role labeling task that is important in extraction of event participants. FrameNet for English is the most developed one [23]. Frames, Frame Elements (FEs), Lexical Units (LU), and valences that expose a frame structure in example sentences are basic elements of the FrameNet.

There are FrameNets for Bulgarian [24] and other languages (German, Danish, and Japanese to name a few). These resources share many mutual features; as all of them are based on the Frame Semantics theory. Manual construction of a FrameNet-like resource is a time-consuming task that is based on parsing and semantic annotation of text corpora. Development of FrameNet for Russian is described in [25]. This resource has a specific structure and contains about 1300 verbs, and it is still far from full coverage of more than 20,000 Russian verbs. Tonelli and Pianta [26] tried to build FrameNet for Italian semiautomatically. Authors populated Italian frames exploiting existing resources: Wikipedia, Wordnet, and English FrameNet. They provide algorithms for translating English FrameNet into Italian as well as annotated corpora (about 50,000 sentences in total).

We propose a method facilitating semiautomatic FrameNet construction for underresourced languages. The method requires GBNC and a thesaurus for a particular language. The following linguistic resources are needed to automate a FrameNet construction:

- a thesaurus (Wordnet or a similar resource);
- a subordination vocabulary;
- a dependency parser and a large text corpus (or the GBNC dataset).

Despite several attempts to develop WordNet for Russian, there is no such resource so far. However, there is a large thesaurus, RuThes-lite, developed in Moscow State University (http://www.dialog-21.ru/digests/dialog2014/materials/pdf/LoukachevitchNV.pdf). The thesaurus has a hierarchical structure similar to Wordnet's structure, but the two resources also have significant differences. We will not describe all features of the thesaurus here and recommend an interested reader to refer to the recent work [27].

We carried out an additional postprocessing step of the GBNC, a conceptual indexing step. In this step if the second word in 2-gram (or the third word in the 3-gram) have “NOUN” PoS-tag, then the word is matched with a concept from the RuThes-lite thesaurus. In the case of multiple matches (e.g., multiple word senses), we exclude the corresponding *n*-gram from the resulting dataset. This preprocessing step resulted in a conceptually enriched dataset (we will refer to it as c-GBNC) that contains the following information for each *n*-gram:

(1) an unambiguous part-of-speech tag and lemma for each word in the *n*-gram;
(2) an unambiguous set of grammatical features (e.g., case, gender, and number) for each word in the *n*-gram;
(3) an unambiguous RuThes-lite concept for the last word in the *n*-gram.

Thus, after this step the dataset is enriched with grammatical and semantic information.

#### 2.4.2. Vocabulary of Semantic Types and Frame Elements Extraction

Frame Elements can be discovered from the c-GBNC dataset using valences of a given verb. But, FEs from different frames may share the same verb and its subordination model. In this case, FEs can be discovered using differences in senses of a subordinate noun. Thus, the next step is building a vocabulary of semantic types and attaching a subordination model of a given verb to one or more semantic types from the vocabulary.

We use top level concepts of the RuThes-lite as a vocabulary of basic semantic types. As the RuThes-lite has too few concepts (such as “Persistent Entity,” “Occurrent Entity,” and “role, place”) at the top level, we consider such distinctions as insufficient. We extended the vocabulary with immediate descendants of the top concepts and obtained about 150 concepts (further we call them “semantic types”). Using a hierarchy of the RuThes-lite, each concept can be mapped to a certain semantic type from the vocabulary.

The main idea behind creating the vocabulary of semantic types is discovering of Frame Elements. We claim that FE may be defined by a unique combination of a verb sense, a case of a subordinated noun (or noun phrase), and the semantic type of the noun. Distinct semantic types will describe differences between FEs sharing the same verb and noun case. Of course, the unique combination should include prepositions if it is the case in a subordinate model of the given verb. This is illustrated in the following examples: ходить  в  одиночестве (to walk alone), ходить  в  шортах (to walk in shorts), and ходить  в  городе (to walk in the city). All three phrases have the same syntactic realization but have different semantic types of subordinate nouns. We discovered FEs for about 10,000 Russian verbs.

## 3. Results and Discussion

We have run few experiments for the proposed methods, that is, extraction of subordination models, discovering FEs and event triggers. We also evaluated FE discovery algorithm on English GBNC dataset, using English FrameNet and Wordnet. We observe a possibility for extension of FrameNet using the FE discovery method.

### 3.1. Extraction of Subordination Models

In order to extract subordination models, we have run two types of SQL-queries, described in the previous section, against the m-GBNC dataset. We have got about 24 thousand rows (one row per verb) from the dependency pair dataset and about 51.5 thousand rows from the 3-gram dataset (a verb + preposition per row). Samples from the resulting subordination models vocabulary are provided in Tables 2 and 3. The normalized weights (representing fraction of corresponding grammatical case) can further be used in modeling a probability for a given verb to superordinate a word in particular case.

The interesting result is that many verbs can superordinate nouns (as direct object) in almost any grammatical form. However, in most rows there is a singe dominating grammatical case (or two). Subordination of preposition is different.

### 3.2. Frame Elements Extraction

In the second experiment we discovered Frame Elements. The total amount of discovered FEs covers about 10,000 verbs, due to elimination of nouns with multiple senses.  Table 4 shows a sample of the result dataset for verb купить (to buy). The sample illustrates both situations when splitting of FE (Locative rows) and merging of FE (genitive and dative rows) are needed. The result dataset of extracted FEs is available http://framenet.s3-website-us-east-1.amazonaws.com/.

In Table 4 we labeled few rows with asterisk. These rows have concepts and semantic types with a distinct meaning. Such heterogeneity in the FE indicates the corresponding combination of verb, preposition, and case covers few semantic roles and needs splitting into two or more roles. That is why a developer of event extraction system should consider semantic type of the participant, not only its syntactic features.

In a separate experiment we evaluated the method of FE extraction in English. For English both GBNC data and and WordNet are available, so we use Wordnet instead of RuThes-lite. We run FE extraction algorithm on the English GBNC dataset and compared results to the English FrameNet. We examined all extracted FEs for the a verb “to buy.'' Each FE that appears in English FrameNet (as of version 1.5) also appears in the extracted dataset. Additionally, our method extracted several FEs that were not described in the FrameNet. For example, consider the following FE:

 “to buy* into* the house, firm, business, company, etc.”

This FE should not be confused with another one “to buy into the idea.” The latter was extracted too, but it has different meaning and should be considered as a part of another frame.

### 3.3. Extraction of Event Triggers

We run two experiments for trigger list acquisition. In the first experiment, starting from two words “купить” (to buy) and “продать” (to sell) the expert selected the following trigger list: (it took about ten minutes): “взять” (to take), “выменять” (to exchange), “обменять” (to exchange), “арендовать” (to rent), “приобрести” (to purchase), “приобретать” (to purchase), “сбыть” (to sell), “достать” (to get), “заложить” (to lease), “отдать” (to give), “передать” (to transfer), “подарить” (to present), “покупать” (to buy), “получать” (to get), “получить” (to get), “променять” (to exchange), “завещать” (to bequeath), “заказать” (to order), “сдать” (to lease), “брать” (to take), “заказывать” (to order), “обменивать” (to barter), “продавать” (to sell), “давать” (to give), “отдавать” (to give), “потерять” (to lose), “выменивать” (to barter), “передавать” (to pass), “дарить” (to give), “закладывать” (to lay), “отчуждать” (to alienate), and “сдавать” (to take).

All of these triggers more or less related to the TRANSFER-OWNERSHIP event from the ACE classification. We compared this list to the RuThes-lite and observed the following. Discovered event triggers are distributed among few hierarchies of the thesaurus, because they have different meaning. Gathering such trigger list will need exploration of almost whole thesaurus following association links, meronymy link, and hyponymy (is-a) links. On the other hand RuThes-lite serves as an excellent starting point for our bootstrap trigger extraction algorithm.

In another experiment we measured quality of the proposed algorithm on a special case of protest and demonstration events. We used the following source set of triggers, proposed in [28], and translated them into Russian:

“демонстрация” (demonstration), “манифестация” (manifestation), “протест” (protest), “митинг” (rally), “акция” (action), “бойкот” (boycott), “забастовка” (strike), “пикет” (picketing), “голодовка” (hunger), “собрание” (gathering), “парад” (parade), “процессия” (procession), “марш” (march), “бунт” (riot), “восстание” (uprising), “мятеж” (mutiny), “беспорядки” (disorder), and “волнение” (unrest).

Then starting with two seed words “митинг” (rally) and “восстание” (uprising), in a single iteration the trigger extraction algorithm collected a list of 39 words that contained 80% of triggers from the source set. Only 29% of words in the list were not related to public manifestation events. Thus, we obtained about 75% of *F*_1_-measure and a set of additional triggers, for instance, “стачка” (strike), “возмущение” (indignation), “заговор” (conspiracy), “революция” (revolution), “сборище” (bunch), “смута” (distemper), “сход” (meeting, gathering), “сходка” (congregation), “съезд” (congress), and “шествие” (cortege, procession).

## 4. Conclusion

The paper presents an overview of state of the art in knowledge event extraction task from Russian texts.

We describe a set of necessary linguistic resources and propose a technology for creation (development) of such resources for Russian and other languages. We faced the problem of manual construction of the following vocabularies that play a central role in event extraction: a vocabulary of event triggers (words that indicate mention of an event in a sentence), a vocabulary of subordination models, and a vocabulary of Frame Elements. Key feature of our technology is using Google Books NGram Corpus in construction of the vocabularies. Experiments in event extraction development system for HP show that a complete vocabulary of event triggers is a bottleneck; a developer cannot account all possible ways of expressing event in language.

In this paper we propose a vocabulary of subordination models. The resulting vocabulary for Russian contains more than 75 thousand units: both pairs (verb + case of subordinated word) and triples (verb + preposition + case of subordinated word). The vocabulary is bigger than others and has information of frequencies of constructions. For more than 10 thousand Russian verbs we developed a vocabulary of Frame Element that forms a basis for frame-based extraction patterns. Each Frame Element describes a pair that related some event trigger to a possible concept from the RuThes-lite ontology.

We also provide baseline for natural language processing: PoS-tagging (with accuracy more than 93%) and parsing (accuracy: 85%). All created resources are available online for noncommercial usage. We proposed a hybrid approach to development of an event extraction system. The approach combines manual work (pattern construction) and automatic corpus-based methods for development of vocabularies for event extraction. The approach reduces efforts needed for traditional manual development of event extraction system. Our future goal is to evaluate the prototype system for event extraction and consequently assess a quality of vocabularies.

## Figures and Tables

**Algorithm 1:**
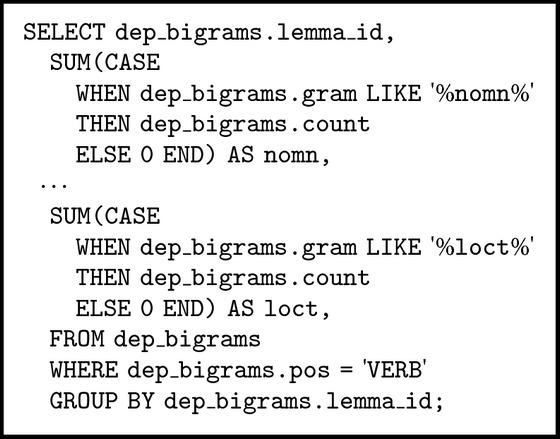


**Table 1:** Amounts of 3 grams with equal PoS-tags.

Conjunction	Noun	Verb	Infn.
ИЛИ (or)	30980	1990	2340
И (and)	492477	54887	30313

**Table 2:** Generated subordination models of direct object for frequent Russian verbs.

Major case	Genitive	Dative	Accusative	Ablative or Instrum.	Infin. form
Dative	0.024	0.905	0.002	0.060	помочь
Dative	0.067	0.679	0.174	0.060	передать
Dative	0.183	0.573	0.057	0.133	сказать
Dative	0.194	0.511	0.252	0.025	дать
Dative	0.171	0.504	0.005	0.296	ответить
Dative	0.192	0.434	0.070	0.166	говорить
Dative	0.163	0.433	0.191	0.041	строить
Dative	0.185	0.433	0.170	0.178	показать
Dative	0.207	0.389	0.174	0.123	писать
Dative	0.299	0.380	0.252	0.046	давать
Dative	0.216	0.377	0.338	0.056	указать
Dative	0.109	0.371	0.323	0.188	доказать
Dative	0.239	0.366	0.046	0.228	судить
Genitive	0.409	0.359	0.127	0.080	делать
Accusative	0.131	0.338	0.352	0.115	изменить
Ablative or Instrum.	0.093	0.292	0.113	0.489	объяснить
Ablative or Instrum.	0.149	0.280	0.006	0.498	действовать
Accusative	0.148	0.271	0.454	0.075	написать
Ablative or Instrum.	0.147	0.264	0.083	0.462	смотреть
Accusative	0.157	0.264	0.377	0.176	представить
Accusative	0.203	0.227	0.397	0.063	оставить
Ablative or Instrum.	0.050	0.209	0.008	0.724	служить
Accusative	0.196	0.198	0.449	0.102	читать
Ablative or Instrum.	0.171	0.190	0.015	0.530	жить
Genitive	0.377	0.188	0.087	0.210	есть

**Table 3:** Generated subordination models of prepositions for verb “купить” (to buy).

Preposition	Major case	Genitive	Dative	Accusative	Ablative or Instrum.	Locative
для (for)	Genitive	1.0	0.0	0.0	0.0	0.0
из (from)	Genitive	1.0	0.0	0.0	0.0	0.0
без (without)	Genitive	1.0	0.0	0.0	0.0	0.0
до (before)	Genitive	1.0	0.0	0.0	0.0	0.0
с (with)	Genitive	0.595	0.0	0.0	0.405	0.0
в (in)	Locative	0.0	0.011	0.068	0.0	0.921
к (to)	Dative	0.0	1.0	0.0	0.0	0.0
на (on)	Locative	0.0	0.049	0.138	0.005	0.808
по (for)	Dative	0.0	1.000	0.0	0.0	0.0
под (under)	Ablative or Instrum.	0.0	0.0	0.0	1.0	0.0

**Table 4:** Frame Elements extracted from GBNC for verb “купить” (to buy).

Case	Preposition	Nouns	RuThes Concepts	Semantic types	Count
Locative	в	киоске, париже, лондоне, среднем^*∗*^, колхозе, магазинах, …	ТОРГОВАЯ ПАЛАТКА, ПАРИЖ, ЛОНДОН, СРЕДНИЙ^*∗*^, АПТЕКА, МАГАЗИН, …	СУБЪЕКТ ДЕЯТЕЛЬНОСТИ, ФИЗИЧЕСКАЯ СУЩНОСТЬ, АБСТРАКТНАЯ^*∗*^ СУЩНОСТЬ^*∗*^ …	15

Locative	на	базаре, рынке, аукционе^*∗*^, вокзале …	РЫНОК, БАЗАР, РЫНОЧНАЯ^*∗*^ ЭКОНОМИКА^*∗*^, АУКЦИОН^*∗*^, ВОКЗАЛ	ФИЗИЧЕСКАЯ, СУЩНОСТЬ, ЗАНЯТИЕ^*∗*^, ДЕЯТЕЛЬНОСТЬ^*∗*^ …	5

Genitive	для	колхоза, жены	КОЛХОЗ, ЖЕНА	СУБЪЕКТ ДЕЯТЕЛЬНОСТИ, ФИЗИЧЕСКАЯ СУЩНОСТЬ	2

Dative	—	сыну, мальчику, правительству, крестьянам, …	СЫН, МАЛЬЧИК, ПРАВИТЕЛЬСТВО, КРЕСТЬЯНИН …	ФИЗИЧЕСКАЯ СУЩНОСТЬ, СОВОКУПНОСТЬ ЛЮДЕЙ, …	23
